# Case Report: A novel MET exon 14 skipping mutation after EGFR-TKI resistance in advanced lung adenocarcinoma and sustained clinical response to savolitinib

**DOI:** 10.3389/fphar.2025.1489696

**Published:** 2025-03-10

**Authors:** Yinyin Xue, Wen Li, Pengfei Li, Kaili Huang, Qinghua Zhou, Qiang Wu

**Affiliations:** ^1^ Department of Radiation Oncology Cancer Center, West China Hospital, Sichuan University, Chengdu, Sichuan, China; ^2^ Lung Cancer Center/Lung Cancer Institute, West China Hospital, Sichuan University, Chengdu, Sichuan, China; ^3^ Department of Medical Oncology Cancer Center, West China Hospital, Sichuan University, Chengdu, Sichuan, China; ^4^ Department of Thoracic Surgery, West China Hospital, Sichuan University, Chengdu, Sichuan, China

**Keywords:** novel MET exon 14 skipping mutation, EGFR-TKI resistance, advanced lung adenocarcinoma, savolitinib, sustained clinical response

## Abstract

**Background:**

The MET proto-oncogene (MET) plays a crucial role as an oncogenic driver gene in non-small cell lung cancer (NSCLC). At present, numerous types of MET exon 14 (METex14) skipping mutation have been identified, but different splice variants often exhibit varying treatment responses. There is currently no standardized treatment approach for rare METex14 mutation after resistance to epidermal growth factor receptor tyrosine kinases inhibitor (EGFR-TKI). Herein, we present for the first time a case of advanced lung adenocarcinoma with a novel METex14 skipping mutation following resistance to EGFR-TKI and subsequent sensitivity to savolitinib. In addition, the patient developed a novel METex14 skipping mutation after EGFR-TKI resistance, which we suspect may be a potential new mechanism of EGFR-TKI resistance that has not been reported.

**Materials and methods:**

We conducted surgical specimen pathology diagnosis and next-generation sequencing (NGS) of peripheral blood to ascertain the patient’s pathological and molecular characteristics.

**Results:**

NGS testing identified a novel METex14 (c.2888-23_2888-8del) skipping mutation in the patient with advanced lung adenocarcinoma who developed resistance to EGFR-TKI, suggesting its potential involvement as one of the mechanisms underlying the resistance to EGFR-TKI. Following administration of savolitinib with a daily dose of 400 mg, the patient exhibited a partial response and achieved progression-free survival (PFS) exceeding 8 months.

**Conclusion:**

The case presents a novel METex14 skipping mutation that emerges subsequent to the progression of advanced lung adenocarcinoma following EGFR-TKI treatment. Importantly, this mutation may serve as one of the mechanisms contributing to resistance against EGFR-TKI and exhibit sensitivity towards savolitinib treatment, providing reference for future similar cases in terms of treatment options.

## Introduction

Epidermal growth factor receptor tyrosine kinase inhibitors (EGFR-TKIs) are the first-line treatment for patients with locally advanced or metastatic EGFR mutation non-small cell lung cancer (NSCLC) (Hanna et al., 2017; Planchard et al., 2018). However, the development of resistance is inevitable in patients receiving EGFR-TKIs. The current mechanism of acquired EGFR-TKIs resistance mainly include the emergence of bypass pathways or histologic alterations, and the Mesenchymal–epithelial transition proto-oncogene (MET) exon 14 (METex14) skipping mutation does not appear to include.

Generally speaking, in approximately 3%–4% of NSCLC patients, the presence of METex14 skipping mutation is observed, which rarely coexists with other known driver mutations in NSCLC, except for MET amplification (Cancer Genome Atlas Research Network, 2014; Baldacci et al., 2018; Frampton et al., 2015; Tong et al., 2016). The METex14 skipping mutation acts as an independent oncogenic driver in NSCLC, and patients with this mutation experience unfavorable prognosis and lower survival rates (Wolf et al., 2020; Yang et al., 2020). Savolitinib is a highly potent and selective MET-TKI that has shown remarkable efficacy and safety in advanced NSCLC patients with METex14 skipping mutation (Lu et al., 2021; Wang et al., 2022). Furthermore, it can be effectively combined with EGFR-TKIs (such as gefitinib, Osimertinib, etc.) to overcome acquired resistance caused by MET alterations (MET amplification or c-MET overexpression), extending the benefits to patients who have previously undergone EGFR-TKIs treatment and experienced disease progression (Sequist et al., 2020; Markham, 2021; Oxnard et al., 2020).

At present, the locations that may lead to METex14 skipping mutation are diverse, warranting further investigation into potential heterogeneity in function and treatment among these distinct MET exon splice variants. It is still unclear whether these newly discovered rare METex14 skipping mutation are sensitive to MET-TKIs. Here, we present a case of advanced lung adenocarcinoma, harboring a novel and rare METex14 skipping mutation, which potentially represents a new mechanism of resistance to EGFR-TKIs.

## Case report

**FIGURE 1:**
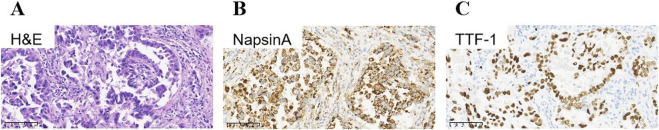
Immunohistochemical staining of primary tumor: Lung adenocarcinoma. **(A)** Hematoxylin-eosin staining of lung adenocarcinoma. Tumor cells were positive for NapsinA **(B)**, and TTF-1 **(C)**. TTF-1, thyroid transcription factor-1.

**FIGURE 2:**
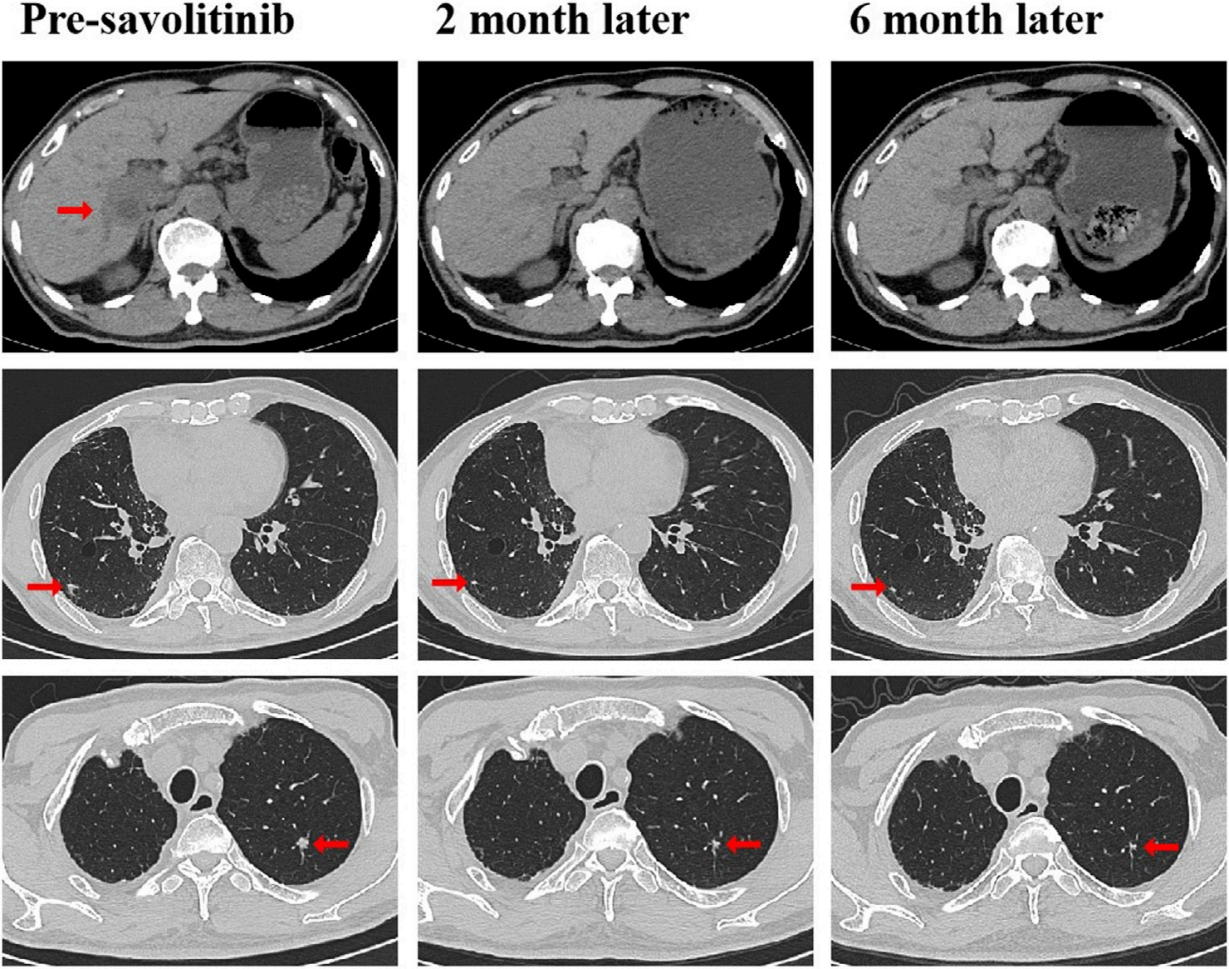
CT scans before and after savolitinib treatment. CT scans at pre-savolitinib showed the presence of liver metastasis and bilateral lung metastases. CT scans after treatment with savolitinib showed partial response in bilateral lung lesions and complete response in liver metastasis. CT, computed tomography.

**FIGURE 3:**
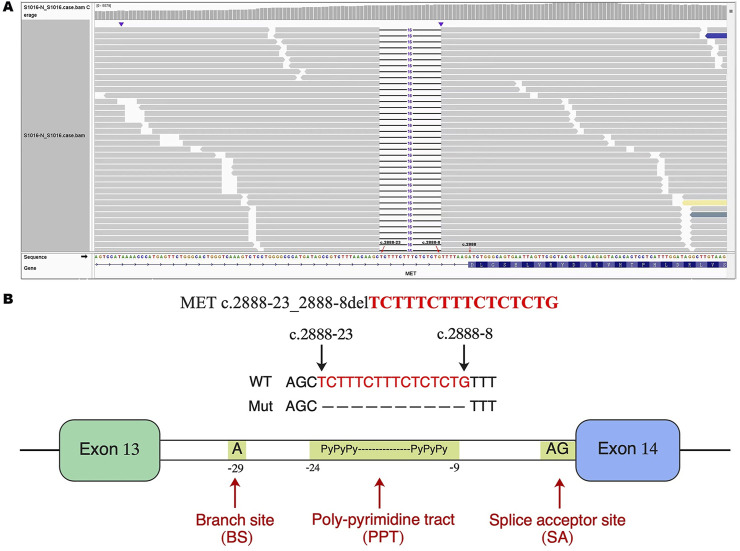
Identification of a novel METex14 skipping mutation by NGS. **(A)** The Integrative Genomics Viewer (IVG) shows the novel METex14 skipping mutation detected by NGS. **(B)** Illustration of METex 14 skipping mutation (the new variant is located near the PPT). METex14, Mesenchymal–epithelial transition (MET) exon 14; NGS, Next-generation sequencing; PPT, poly-pyrimidine tract.

**FIGURE 4:**
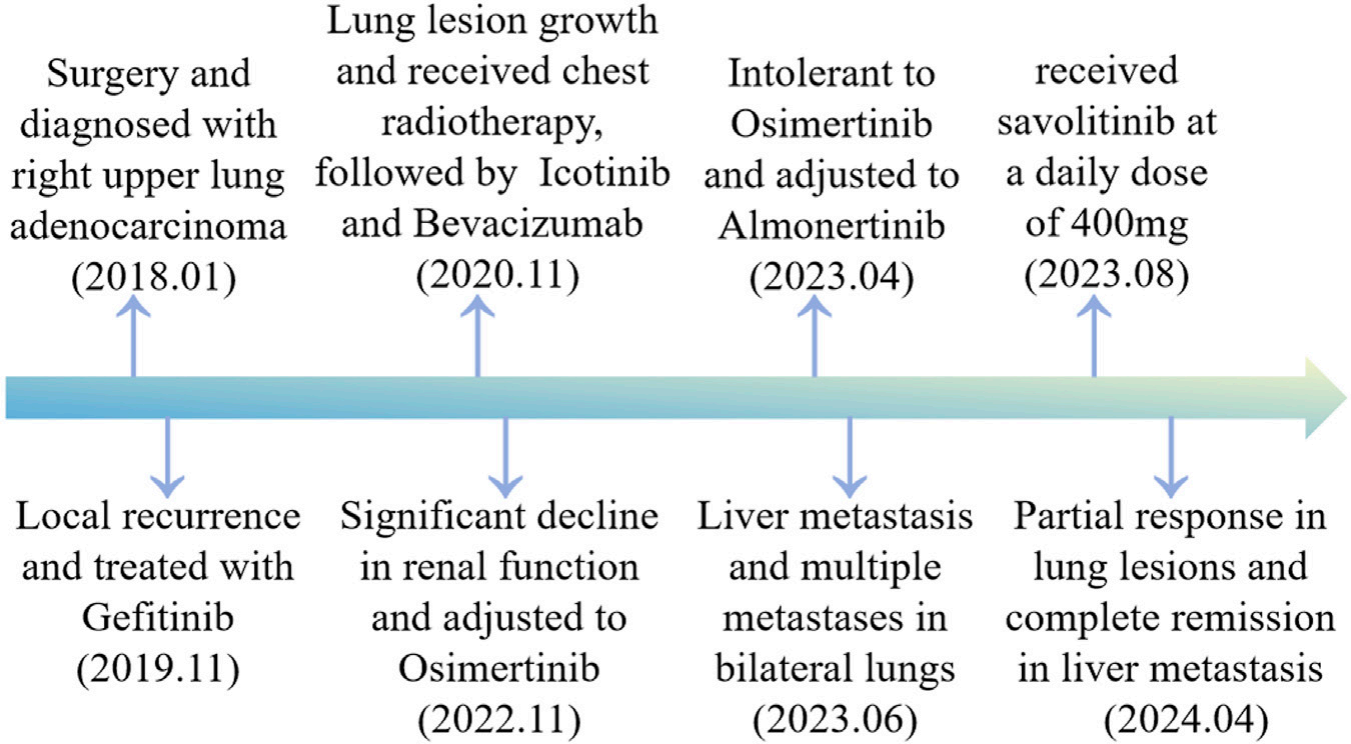
Timeline scheme of major clinical event of the patient since diagnosis.

A 79-year-old non-smoking man visited the hospital because of a 5-month history of cough with sputum. The patient was previously in good health, without hypertension and diabetes. A computed tomography (CT) scan revealed a nodule in the right upper lung (the diameter is approximately 1.2 cm), without bone, liver, kidney, or brain metastases. On 9 January 2018, the patient underwent right upper lobectomy plus systematic lymph node dissection and was diagnosed with right upper lung adenocarcinoma (stage IA, pT1N0M0) as the immunohistochemical (IHC) staining indicated the tumor was positive for Napsin A and thyroid transcription factor-1 (TTF-1) (Figure 1). The gene testing of the patient’s pathological tissue revealed the presence of epidermal growth factor receptor (EGFR) exon 21 L858R point mutation. The patient did not receive postoperative adjuvant therapy and underwent regular follow-up. In November 2019, a chest CT scan revealed local recurrence of the residual right upper lobe of the lung. Subsequently, the patient initiated gefitinib treatment at a daily dose of 250 mg. Four months later, the patient stopped taking gefitinib due to progressive lesion growth and then the patient received chest radiotherapy, followed by continuous treatment with a combination of Icotinib and Bevacizumab in November 2020. Due to a significant decline in renal function (serum creatinine level:123umol/L; estimated glomerular filtration rate (eGFR):48.1 mL/min), Icotinib and Bevacizumab were discontinued in November 2022, and the patient was subsequently switched to Osimertinib. In April 2023, the patient’s chest CT revealed interstitial changes in the lungs, which led to the consideration of possible intolerance to Osimertinib, and the treatment was adjusted to Almonertinib. In June 2023, a CT scan showed bilateral lung metastases and a growth of the tumor in the lower segment of the right posterior lobe of the liver, indicating disease progression (Figure 2), then the peripheral blood next-generation sequencing (NGS) testing was conducted, and the NGS testing revealed a novel METex14 skipping mutation (c.2888–23_2888-8delTCTTTCTTTCTCTCTG IVS13, 1.0%), accompanied by a TP53 mutation (p.Y163C, 0.1%) (Figure 3). Combination therapy with Almonertinib and savolitinib were recommended, but the patient chose to receive savolitinib alone at a daily dose of 400 mg after considering the cost. Fortunately, after a 2-month period of treatment, the patient had a partial response in lung lesions and complete response in liver metastasis (Figure 2). As of the time of writing this manuscript, the patient is still taking savolitinib with a progression-free survival (PFS) over 8 months and no significant adverse events have been observed so far. The patient’s treatment process is shown in Figure 4.

## Discussion

The novel METex14 skipping mutation (c.2888-23_2888-8del) reported here is located near the poly-pyrimidine tract (PPT), which may lead to the skipping mutation in exon 14. To our knowledge, this study presents the first report of a novel METex14 skipping mutation observed in NSCLC patients following resistance to EGFR-TKIs treatment. We propose that this mutation may serve as a potential mechanism of resistance to EGFR-TKIs, which has not been previously reported. In addition, this study provides the first clinical evidence demonstrating that savolitinib effectively reverses acquired resistance to EGFR-TKIs driven by novel METex14 skipping mutation in patients with advanced lung adenocarcinoma harboring EGFR mutations, thereby yielding long-term PFS benefits.

The bypassing of activation mediated by the MET signaling pathway is a crucial mechanism contributing to resistance against EGFR-TKI, which can manifest as gene-level amplification or protein-level overexpression (Reis et al., 2018; Wood et al., 2021). The effective approach for managing acquired resistance to EGFR-TKIs driven by MET is combination therapy involving EGFR-TKIs and MET-TKIs (Oxnard et al., 2020; York et al., 2017; Gainor et al., 2016; Wu et al., 2018). Currently, savolitinib is primarily employed as a monotherapy for advanced NSCLC with METex14 skipping mutation, as well as in combination with EGFR-TKIs to overcome acquired resistance driven by MET to EGFR-TKIs (Sequist et al., 2020; Markham, 2021; Oxnard et al., 2020; Yu et al., 2024; Lee et al., 2023). However, the resistance mechanisms acquired to EGFR-TKIs driven by MET do not appear to involve METex14 skipping mutation. Our case report showed that the patient developed a new METex14 skipping mutation after acquiring resistance to EGFR-TKIs. We suggested combining savolitinib with the current EGFR-TKI treatment, but the patient chose to only use savolitinib alone and still experienced a significant improvement in PFS. Therefore, in such cases, it may be advisable to assess the viability of MET-TKI monotherapy, considering the patient’s physical tolerance, family financial circumstances, and personal willingness.

The presence of TP53 gene mutations is frequently observed in NSCLC with METex14 skipping mutation (Frampton et al., 2015). While the coexistence of TP53 mutations has been associated with reduced efficacy of EGFR-TKIs in NSCLC, there is no evidence to suggest that alterations in TP53 impact the effectiveness of MET-TKIs (Canale et al., 2017; Fujino et al., 2021). The optimal diagnostic method for METex14 skipping mutation is based on NGS of DNA or RNA (Kim et al., 2019). Based on the NGS analysis, our case report demonstrated the concurrent presence of METex14 skipping mutation and TP53 mutation in the patient, with a PFS exceeding 8 months. Further investigation is necessary to understand the impact of TP53 coexistence on the effectiveness of MET-TKIs.

Currently, the indications for administering MET-TKIs to rare MET exon splice variants are still a subject of controversy due to the heterogeneous response observed among them. Hence, it is essential to utilize NGS for a thorough identification of MET exon splicing variants, aiming to optimize treatment strategies and improve patient survival with MET-TKIs. The novel METex14 skipping mutation, detected by NGS in this case, showed a positive response to savolitinib, resulting in a PFS of over 8 months. Therefore, we propose that this mutation is sensitive to MET-TKIs. Until the submission of the manuscript, the patient is still undergoing savolitinib treatment, and we will continue to monitor the therapeutic efficacy.

In summary, our case report presented a lung adenocarcinoma patient who developed a novel METex14 skipping mutation (c.2888-23_2888-8del) after acquiring resistance to EGFR-TKIs treatment and exhibited clinical benefits from savolitinib therapy. Moreover, we proposed that this newly identified METex14 skipping mutation may represent an emerging mechanism of acquired resistance to EGFR-TKI treatment, warranting further investigation for validation.

## Data Availability

The original contributions presented in the study are included in the article/supplementary material, further inquiries can be directed to the corresponding author.
